# Why structure matters

**DOI:** 10.7554/eLife.45380

**Published:** 2019-03-21

**Authors:** Nick Barton, Joachim Hermisson, Magnus Nordborg

**Affiliations:** 1 IST AustriaKlosterneuburgAustria; 2 Department of MathematicsUniversity of ViennaViennaAustria; 3 Max F. Perutz LaboratoriesUniversity of ViennaViennaAustria; 4 Gregor Mendel InstituteAustrian Academy of Sciences, Vienna BioCenterViennaAustria

**Keywords:** polygenic adaptation, GWAS, population structure, population genetics, quantitative genetics, selection for human height, Human

## Abstract

Great care is needed when interpreting claims about the genetic basis of human variation based on data from genome-wide association studies.

**Related research article** Sohail M, Maier RM, Ganna A, Bloemendal A, Martin AR, Turchin MC, Chiang CWK, Hirschhorn J, Daly MJ, Patterson N, Neale B, Mathieson I, Reich D, Sunyaev SR. 2019. Polygenic adaptation on height is overestimated due to uncorrected stratification in genome-wide association studies. *eLife* **8**:e39702. doi: 10.7554/eLife.39702

**Related research article** Berg JJ, Harpak A, Sinnott-Armstrong N, Joergensen AM, Mostafavi H, Field Y, Boyle EA, Zhang X, Racimo F, Pritchard JK, Coop G. 2019. Reduced signal for polygenic adaptation of height in UK Biobank. *eLife* **8**:e39725. doi: 10.7554/eLife.39725

Human height is the classic example of a quantitative trait: its distribution is continuous, presumably because it is influenced by variation at a very large number of genes, most with a small effect (Fisher, 1918). Yet height is also strongly affected by the environment: average height in many countries increased during the last century and the children of immigrants are often taller than relatives in their country of origin – in both cases presumably due to changing diet and other environmental factors (Cavalli-Sforza and Bodmer, 1971; Grasgruber et al., 2016; NCD Risk Factor Collaboration, 2016). This makes it very difficult to determine the cause of geographic patterns for height, such as the ‘latitudinal cline’ seen in Europe (Figure 1).

**Figure 1.**
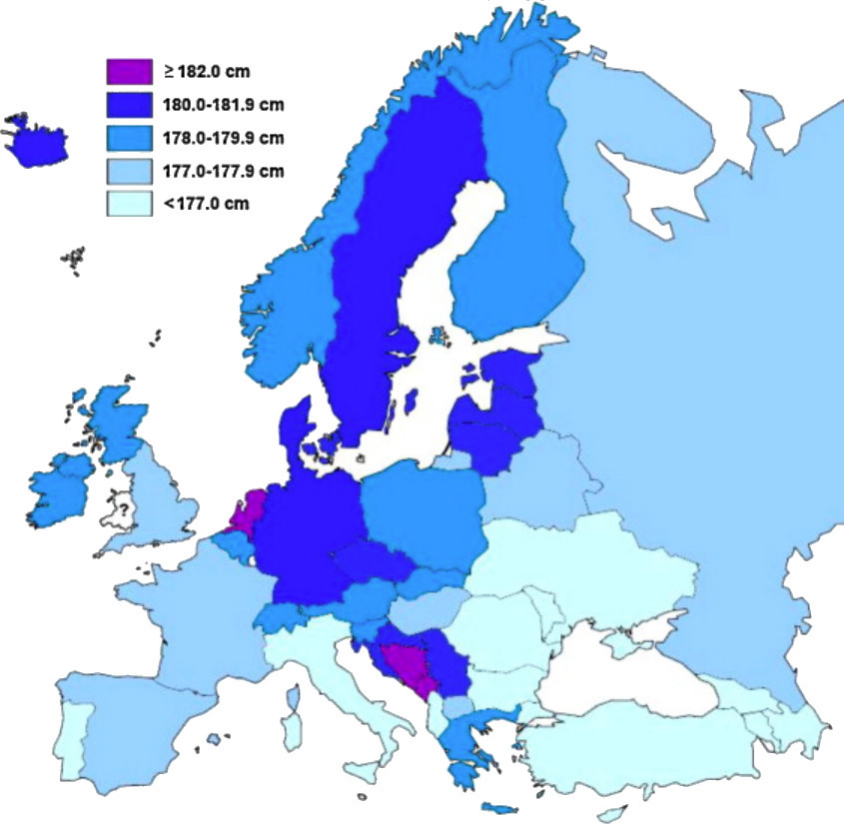
Distribution of average male height in Europe, calculated from studies performed between 1999–2013. In general, southern Europeans tend to be shorter than northern Europeans. Image reproduced from Grasgruber et al., 2014 (CC BY 3.0).

Are such patterns caused by environmental or genetic differences – or by a complex combination of both? And to the extent that genetic differences are involved, do they reflect selection or simply random history? A number of recent papers have relied on so-called Genome-Wide Association Studies (GWAS) to address these questions, and reported strong evidence for both genetics and selection. Now, in eLife, two papers – one by Jeremy Berg, Arbel Harpak, Nasa Sinnott-Armstrong and colleagues (Berg et al., 2019); the other by Mashaal Sohail, Robert Maier and colleagues (Sohail et al., 2019) – independently reject these conclusions. Even more importantly, they identify problems with GWAS that have broader implications for human genetics.

As the name suggests, GWAS scan the genome for variants – typically single nucleotide polymorphisms (SNPs) – that are associated with a particular condition or trait (phenotype). The first GWAS for height found a small number of SNPs that jointly explained only a tiny fraction of the variation. Because this was in contrast with the high heritability seen in twin studies, it was dubbed ‘the missing heritability problem’ (reviewed in Yang et al., 2010). It was suggested that the problem was simply due to a lack of statistical power to detect polymorphisms of small effect. Subsequent studies with larger sample sizes have supported this explanation: more and more loci have been identified although most of the variation remains ‘unmappable’, presumably because sample sizes on the order of a million are still not large enough (Yengo et al., 2018).

One way in which the unmappable component of genetic variation can be included in a statistical measure is via so-called polygenic scores. These scores sum the estimated contributions to the trait across many SNPs, including those whose effects, on their own, are not statistically significant. Polygenic scores thus represent a shift from the goal of identifying major genes to predicting phenotype from genotype. Originally designed for plant and animal breeding purposes, polygenic scores can, in principle, also be used to study the genetic basis of differences between individuals and groups.

This, however, requires accurate and unbiased estimation of the effects of all SNPs included in the score, which is difficult in a structured (non-homogeneous) population when environmental differences cannot be controlled. To see why this is a problem, consider the classic example of chopstick-eating skills (Lander and Schork, 1994). While there surely are genetic variants affecting our ability to handle chopsticks, most of the variation for this trait across the globe is due to environmental differences (cultural background), and a GWAS would mostly identify variants that had nothing to do with chopstick skills, but simply happened to differ in frequency between East Asia and the rest of the world.

Several methods for dealing with this problem have been proposed. When a GWAS is carried out to identify major genes, it is relatively simple to avoid false positives by eliminating associations outside major loci regardless of whether they are due to population structure confounding or an unmappable polygenic background (Vilhjálmsson and Nordborg, 2013). However, if the goal is to make predictions, or to understand differences among populations (such as the latitudinal cline in height), we need accurate and unbiased estimates for all SNPs. Accomplishing this is extremely challenging, and it is also difficult to know whether one has succeeded.

One possibility is to compare the population estimates with estimates taken from sibling data, which should be relatively unbiased by environmental differences. In one of many examples of this, Robinson et al. used data from the GIANT Consortium (Wood et al., 2014) together with sibling data to estimate that genetic variation contributes significantly to height variation across Europe (Robinson et al., 2015). They also argued that selection must have occurred, because the differences were too large to have arisen by chance. Using estimated effect sizes provided by Robinson et al., a more sophisticated analysis by Field et al. found extremely strong evidence for selection for height across Europe (p=10^−74^; Field et al., 2016). Several other studies reached the same conclusion based on the GIANT data (reviewed in Berg et al., 2019; Sohail et al., 2019).

Berg et al. (who are based at Columbia University, Stanford University, UC Davis and the University of Copenhagen) and Sohail et al. (who are based at Harvard Medical School, the Broad Institute, and other institutes in the US, Finland and Sweden) now re-examine these conclusions using the recently released data from the UK Biobank (Sudlow et al., 2015). Estimating effect sizes from these data allows possible biases due to population structure confounding to be investigated, because the UK Biobank data comes from a (supposedly) more homogenous population than the GIANT data.

Using these new estimates, Berg et al. and Sohail et al. independently found that evidence for selection vanishes – along with evidence for a genetic cline in height across Europe. Instead, they show that the previously published results were due to the cumulative effects of slight biases in the effect-size estimates in the GIANT data. Surprisingly, they also found evidence for confounding in the sibling data used as a control by Robinson et al. and Field et al. This turned out to be due to a technical error in the data distributed by Robinson et al. after they published their paper.

This means we still do not know whether genetics and selection are responsible for the pattern of height differences seen across Europe. That genetics plays a major role in height differences between individuals is not in doubt, and it is also clear that the signal from GWAS is mostly real. The issue is that there is no perfect way to control for complex population structure and environmental heterogeneity. Biases at individual loci may be tiny, but they become highly significant when summed across thousands of loci – as is done in polygenic scores. Standard methods to control for these biases, such as principal component analysis, may work well in simulations but are often insufficient when confronted with real data. Importantly, no natural population is unstructured: indeed, even the data in the UK Biobank seems to contain significant structure (Haworth et al., 2019).

Berg et al. and Sohail et al. demonstrate the potential for population structure to create spurious results, especially when using methods that rely on large numbers of small effects, such as polygenic scores. Caution is clearly needed when interpreting and using the results of such studies. For clinical predictions, risks must be weighed against benefits (Rosenberg et al., 2019). In some cases, such as recommendations for more frequent medical checkups for patients found at higher ‘genetic’ risk of a condition, it may not matter greatly whether predictors are confounded as long as they work. By contrast, the results of behavioral studies of traits such as IQ and educational attainment (Plomin and von Stumm, 2018) must be presented carefully, because while the benefits are far from obvious, the risks of such results being misinterpreted and misused are quite clear. The problem is worsened by the tendency of popular media to ignore caveats and uncertainties of estimates.

Finally, although quantitative genetics has proved highly successful in plant and animal breeding, it should be remembered that this success has been based on large pedigrees, well-controlled environments, and short-term prediction. When these methods have been applied to natural populations, even the most basic predictions fail, in large part due to poorly understood environmental factors (Charmantier et al., 2014). Natural populations are never homogeneous, and it is therefore misleading to imply there is a qualitative difference between ‘within-population’ and ‘between-population’ comparisons – as was recently done in connection with James Watson’s statements about race and IQ (Harmon, 2019). With respect to confounding by population structure, the key qualitative difference is between controlling the environment experimentally, and not doing so. Once we leave an experimental setting, we are effectively skating on thin ice, and whether the ice will hold depends on how far out we skate.

## References

[bib1] Berg JJ, Harpak A, Sinnott-Armstrong N, Joergensen AM, Mostafavi H, Field Y, Boyle EA, Zhang X, Racimo F, Pritchard JK, Coop G (2019). Reduced signal for polygenic adaptation of height in UK Biobank. eLife.

[bib2] Cavalli-Sforza LL, Bodmer WF (1971). The Genetics of Human Populations.

[bib3] Charmantier A, Garant D, Kruuk LEB (2014). Quantitative Genetics in the Wild.

[bib4] Field Y, Boyle EA, Telis N, Gao Z, Gaulton KJ, Golan D, Yengo L, Rocheleau G, Froguel P, McCarthy MI, Pritchard JK (2016). Detection of human adaptation during the past 2000 years. Science.

[bib5] Fisher RA (1918). XV.—The correlation between relatives on the supposition of Mendelian inheritance. Transactions of the Royal Society of Edinburgh.

[bib6] Grasgruber P, Cacek J, Kalina T, Sebera M (2014). The role of nutrition and genetics as key determinants of the positive height trend. Economics & Human Biology.

[bib7] Grasgruber P, Sebera M, Hrazdíra E, Cacek J, Kalina T (2016). Major correlates of male height: A study of 105 countries. Economics & Human Biology.

[bib8] Harmon A (2019). James Watson had a chance to salvage his reputation on race. He made things worse. The New York Times.

[bib9] Haworth S, Mitchell R, Corbin L, Wade KH, Dudding T, Budu-Aggrey A, Carslake D, Hemani G, Paternoster L, Smith GD, Davies N, Lawson DJ, J Timpson N (2019). Apparent latent structure within the UK Biobank sample has implications for epidemiological analysis. Nature Communications.

[bib10] Lander ES, Schork NJ (1994). Genetic dissection of complex traits. Science.

[bib11] NCD Risk Factor Collaboration (2016). A century of trends in adult human height. eLife.

[bib12] Plomin R, von Stumm S (2018). The new genetics of intelligence. Nature Reviews Genetics.

[bib13] Robinson MR, Hemani G, Medina-Gomez C, Mezzavilla M, Esko T, Shakhbazov K, Powell JE, Vinkhuyzen A, Berndt SI, Gustafsson S, Justice AE, Kahali B, Locke AE, Pers TH, Vedantam S, Wood AR, van Rheenen W, Andreassen OA, Gasparini P, Metspalu A, Berg LH, Veldink JH, Rivadeneira F, Werge TM, Abecasis GR, Boomsma DI, Chasman DI, de Geus EJ, Frayling TM, Hirschhorn JN, Hottenga JJ, Ingelsson E, Loos RJ, Magnusson PK, Martin NG, Montgomery GW, North KE, Pedersen NL, Spector TD, Speliotes EK, Goddard ME, Yang J, Visscher PM (2015). Population genetic differentiation of height and body mass index across Europe. Nature Genetics.

[bib14] Rosenberg NA, Edge MD, Pritchard JK, Feldman MW (2019). Interpreting polygenic scores, polygenic adaptation, and human phenotypic differences. Evolution, Medicine, and Public Health.

[bib15] Sohail M, Maier RM, Ganna A, Bloemendal A, Martin AR, Turchin MC, Chiang CW, Hirschhorn J, Daly MJ, Patterson N, Neale B, Mathieson I, Reich D, Sunyaev SR (2019). Polygenic adaptation on height is overestimated due to uncorrected stratification in genome-wide association studies. eLife.

[bib16] Sudlow C, Gallacher J, Allen N, Beral V, Burton P, Danesh J, Downey P, Elliott P, Green J, Landray M, Liu B, Matthews P, Ong G, Pell J, Silman A, Young A, Sprosen T, Peakman T, Collins R (2015). UK Biobank: an open access resource for identifying the causes of a wide range of complex diseases of middle and old age. PLOS Medicine.

[bib17] Vilhjálmsson BJ, Nordborg M (2013). The nature of confounding in genome-wide association studies. Nature Reviews Genetics.

[bib18] Wood AR, Esko T, Yang J, Vedantam S, Pers TH, Gustafsson S, Chu AY, Estrada K, Luan J, Kutalik Z, Amin N, Buchkovich ML, Croteau-Chonka DC, Day FR, Duan Y, Fall T, Fehrmann R, Ferreira T, Jackson AU, Karjalainen J, Lo KS, Locke AE, Mägi R, Mihailov E, Porcu E, Randall JC, Scherag A, Vinkhuyzen AA, Westra HJ, Winkler TW, Workalemahu T, Zhao JH, Absher D, Albrecht E, Anderson D, Baron J, Beekman M, Demirkan A, Ehret GB, Feenstra B, Feitosa MF, Fischer K, Fraser RM, Goel A, Gong J, Justice AE, Kanoni S, Kleber ME, Kristiansson K, Lim U, Lotay V, Lui JC, Mangino M, Mateo Leach I, Medina-Gomez C, Nalls MA, Nyholt DR, Palmer CD, Pasko D, Pechlivanis S, Prokopenko I, Ried JS, Ripke S, Shungin D, Stancáková A, Strawbridge RJ, Sung YJ, Tanaka T, Teumer A, Trompet S, van der Laan SW, van Setten J, Van Vliet-Ostaptchouk JV, Wang Z, Yengo L, Zhang W, Afzal U, Arnlöv J, Arscott GM, Bandinelli S, Barrett A, Bellis C, Bennett AJ, Berne C, Blüher M, Bolton JL, Böttcher Y, Boyd HA, Bruinenberg M, Buckley BM, Buyske S, Caspersen IH, Chines PS, Clarke R, Claudi-Boehm S, Cooper M, Daw EW, De Jong PA, Deelen J, Delgado G, Denny JC, Dhonukshe-Rutten R, Dimitriou M, Doney AS, Dörr M, Eklund N, Eury E, Folkersen L, Garcia ME, Geller F, Giedraitis V, Go AS, Grallert H, Grammer TB, Gräßler J, Grönberg H, de Groot LC, Groves CJ, Haessler J, Hall P, Haller T, Hallmans G, Hannemann A, Hartman CA, Hassinen M, Hayward C, Heard-Costa NL, Helmer Q, Hemani G, Henders AK, Hillege HL, Hlatky MA, Hoffmann W, Hoffmann P, Holmen O, Houwing-Duistermaat JJ, Illig T, Isaacs A, James AL, Jeff J, Johansen B, Johansson Å, Jolley J, Juliusdottir T, Junttila J, Kho AN, Kinnunen L, Klopp N, Kocher T, Kratzer W, Lichtner P, Lind L, Lindström J, Lobbens S, Lorentzon M, Lu Y, Lyssenko V, Magnusson PK, Mahajan A, Maillard M, McArdle WL, McKenzie CA, McLachlan S, McLaren PJ, Menni C, Merger S, Milani L, Moayyeri A, Monda KL, Morken MA, Müller G, Müller-Nurasyid M, Musk AW, Narisu N, Nauck M, Nolte IM, Nöthen MM, Oozageer L, Pilz S, Rayner NW, Renstrom F, Robertson NR, Rose LM, Roussel R, Sanna S, Scharnagl H, Scholtens S, Schumacher FR, Schunkert H, Scott RA, Sehmi J, Seufferlein T, Shi J, Silventoinen K, Smit JH, Smith AV, Smolonska J, Stanton AV, Stirrups K, Stott DJ, Stringham HM, Sundström J, Swertz MA, Syvänen AC, Tayo BO, Thorleifsson G, Tyrer JP, van Dijk S, van Schoor NM, van der Velde N, van Heemst D, van Oort FV, Vermeulen SH, Verweij N, Vonk JM, Waite LL, Waldenberger M, Wennauer R, Wilkens LR, Willenborg C, Wilsgaard T, Wojczynski MK, Wong A, Wright AF, Zhang Q, Arveiler D, Bakker SJ, Beilby J, Bergman RN, Bergmann S, Biffar R, Blangero J, Boomsma DI, Bornstein SR, Bovet P, Brambilla P, Brown MJ, Campbell H, Caulfield MJ, Chakravarti A, Collins R, Collins FS, Crawford DC, Cupples LA, Danesh J, de Faire U, den Ruijter HM, Erbel R, Erdmann J, Eriksson JG, Farrall M, Ferrannini E, Ferrières J, Ford I, Forouhi NG, Forrester T, Gansevoort RT, Gejman PV, Gieger C, Golay A, Gottesman O, Gudnason V, Gyllensten U, Haas DW, Hall AS, Harris TB, Hattersley AT, Heath AC, Hengstenberg C, Hicks AA, Hindorff LA, Hingorani AD, Hofman A, Hovingh GK, Humphries SE, Hunt SC, Hypponen E, Jacobs KB, Jarvelin MR, Jousilahti P, Jula AM, Kaprio J, Kastelein JJ, Kayser M, Kee F, Keinanen-Kiukaanniemi SM, Kiemeney LA, Kooner JS, Kooperberg C, Koskinen S, Kovacs P, Kraja AT, Kumari M, Kuusisto J, Lakka TA, Langenberg C, Le Marchand L, Lehtimäki T, Lupoli S, Madden PA, Männistö S, Manunta P, Marette A, Matise TC, McKnight B, Meitinger T, Moll FL, Montgomery GW, Morris AD, Morris AP, Murray JC, Nelis M, Ohlsson C, Oldehinkel AJ, Ong KK, Ouwehand WH, Pasterkamp G, Peters A, Pramstaller PP, Price JF, Qi L, Raitakari OT, Rankinen T, Rao DC, Rice TK, Ritchie M, Rudan I, Salomaa V, Samani NJ, Saramies J, Sarzynski MA, Schwarz PE, Sebert S, Sever P, Shuldiner AR, Sinisalo J, Steinthorsdottir V, Stolk RP, Tardif JC, Tönjes A, Tremblay A, Tremoli E, Virtamo J, Vohl MC, Amouyel P, Asselbergs FW, Assimes TL, Bochud M, Boehm BO, Boerwinkle E, Bottinger EP, Bouchard C, Cauchi S, Chambers JC, Chanock SJ, Cooper RS, de Bakker PI, Dedoussis G, Ferrucci L, Franks PW, Froguel P, Groop LC, Haiman CA, Hamsten A, Hayes MG, Hui J, Hunter DJ, Hveem K, Jukema JW, Kaplan RC, Kivimaki M, Kuh D, Laakso M, Liu Y, Martin NG, März W, Melbye M, Moebus S, Munroe PB, Njølstad I, Oostra BA, Palmer CN, Pedersen NL, Perola M, Pérusse L, Peters U, Powell JE, Power C, Quertermous T, Rauramaa R, Reinmaa E, Ridker PM, Rivadeneira F, Rotter JI, Saaristo TE, Saleheen D, Schlessinger D, Slagboom PE, Snieder H, Spector TD, Strauch K, Stumvoll M, Tuomilehto J, Uusitupa M, van der Harst P, Völzke H, Walker M, Wareham NJ, Watkins H, Wichmann HE, Wilson JF, Zanen P, Deloukas P, Heid IM, Lindgren CM, Mohlke KL, Speliotes EK, Thorsteinsdottir U, Barroso I, Fox CS, North KE, Strachan DP, Beckmann JS, Berndt SI, Boehnke M, Borecki IB, McCarthy MI, Metspalu A, Stefansson K, Uitterlinden AG, van Duijn CM, Franke L, Willer CJ, Price AL, Lettre G, Loos RJ, Weedon MN, Ingelsson E, O'Connell JR, Abecasis GR, Chasman DI, Goddard ME, Visscher PM, Hirschhorn JN, Frayling TM, Electronic Medical Records and Genomics (eMEMERGEGE) Consortium MIGen Consortium PAGEGE Consortium LifeLines Cohort Study (2014). Defining the role of common variation in the genomic and biological architecture of adult human height. Nature Genetics.

[bib19] Yang J, Benyamin B, McEvoy BP, Gordon S, Henders AK, Nyholt DR, Madden PA, Heath AC, Martin NG, Montgomery GW, Goddard ME, Visscher PM (2010). Common SNPs explain a large proportion of the heritability for human height. Nature Genetics.

[bib20] Yengo L, Sidorenko J, Kemper KE, Zheng Z, Wood AR, Weedon MN, Frayling TM (2018). Meta-analysis of genome-wide association studies for height and body mass index in ~700,000 individuals of European ancestry. bioRxiv.

